# Multi-Omics Analysis Provides Insights into the Key Regulatory Pathways of Energy Metabolism in GIFT Under Salinity Stress

**DOI:** 10.3390/vetsci13010105

**Published:** 2026-01-21

**Authors:** Yumeng Zhang, Binglin Chen, Dayu Li, Zhiying Zou, Jinglin Zhu, Jie Yu, Hong Yang, Wei Xiao

**Affiliations:** 1 Wuxi Fisheries College, Nanjing Agricultural University, Wuxi 214081, China; zhangyumengpj@foxmail.com (Y.Z.); zhujinglin@ffrc.cn (J.Z.);; 2 Freshwater Fisheries Research Center, Chinese Academy of Fishery Sciences, Wuxi 214081, China; 3 College of Fisheries and Life Science, Dalian Ocean University, Dalian 116000, China

**Keywords:** GIFT, salinity stress, multi-omics analysis, energy reallocation

## Abstract

Salinity stress represents a critical environmental constraint that significantly limits the development of tilapia aquaculture in brackish water environments. Its substantial impacts on fundamental physiological processes in fish, particularly osmotic balance, energy metabolism, and antioxidant defense mechanisms, have become a major scientific concern in aquaculture research. To systematically elucidate the molecular mechanisms underlying the response of genetically improved farmed tilapia (*Oreochromis niloticus*) to salinity stress and to test the hypothesis that it adapts through metabolic reprogramming for energy reallocation under such conditions, this study employed an integrated transcriptomic and metabolomic approach. Through a rigorously controlled experimental design with freshwater (0‰) as the control group and brackish water (24‰) as the experimental group, we conducted a comprehensive analysis of dynamic changes in gene expression profiles and metabolite spectra in the liver tissues of experimental fish. The study yielded the following key findings: First, salinity stress significantly suppressed growth performance indicators, including body weight and length, while simultaneously inducing extensive transcriptomic restructuring and profound metabolic remodeling in liver tissue. A total of 1529 differentially expressed genes (including 399 up-regulated and 1130 down-regulated genes) and 127 significantly differential metabolites were identified. Second, the organism achieved strategic reallocation of energy resources through coordinated suppression of multiple energy-consuming anabolic pathways, particularly steroid biosynthesis and fatty acid metabolism, with the remarkable down-regulation of *Fasn*, a key gene in the fatty acid synthesis pathway, being especially prominent. Energy-sensing and metabolic homeostasis regulatory networks played a central coordinating role in this process, guiding the organism through metabolic reprogramming by regulating downstream metabolic nodes. From a multi-omics integrative perspective, this study provides in-depth insights into the sophisticated metabolic remodeling and energy allocation strategies employed by GIFT to cope with salinity stress. These findings, particularly the suppression of fatty acid biosynthesis and the reprogramming of glycolysis/gluconeogenesis pathways, not only elucidate the molecular mechanisms by which teleosts achieve environmental adaptation through energy reallocation, but also provide actionable molecular targets for the selective breeding of salinity-resilient tilapia strains.

## Simple Summary

Salinity stress poses a major challenge to tilapia farming, particularly as the industry increasingly explores the use of brackish water. This study aimed to investigate the internal response mechanisms of genetically improved farmed tilapia (GIFT) when transferred from freshwater to saline environments. By analyzing changes in genes and small molecules in the fish liver, we found that high-salinity conditions slow fish growth. In response, the fish significantly alter their energy utilization strategies: they actively reduce energy expenditure on the synthesis of fats and steroid hormones and adjust sugar metabolism, thereby redirecting energy toward the critical and energy-intensive task of maintaining internal salt-water balance. These findings reveal the molecular strategies tilapia employ to cope with salt stress. This research provides insights for breeding new tilapia varieties with enhanced salt tolerance, contributing to more sustainable and productive aquaculture across a wider range of aquatic environments.

## 1. Introduction

Salinity is a key environmental factor influencing the survival [1], growth, and distribution of aquatic organisms, as well as a major abiotic stressor in aquaculture. Fluctuations in ambient salinity trigger a series of complex physiological stress responses in fish to maintain homeostasis, involving critical biological processes such as osmoregulation, energy metabolism, antioxidant defense, and immune response [2]. As global aquaculture faces pressures from saltwater intrusion [3], freshwater resource competition [4], and land constraints [5], understanding how economically important species respond to salinity stress becomes increasingly urgent for sustainable industry development. Tilapia, particularly the Nile tilapia (*Oreochromis niloticus*), represents one of the most globally significant aquaculture species [6], renowned for its fast growth rate [7], strong adaptability [8], and high nutritional value [9], ranking as the second most farmed fish worldwide [9]. Traditionally farmed in freshwater environments [10], tilapia’s potential for expansion into brackish [11] and marine environments [12] offers a promising solution to resource challenges. It is noteworthy that salinity tolerance varies significantly among different species within the genus Oreochromis: both the Mozambique tilapia *(O. mossambicus*) and the blue tilapia (*O. aureus*) exhibit strong euryhaline adaptability [13], with the former particularly capable of completing its entire life cycle in full seawater environments, and its molecular mechanisms of osmoregulation have been extensively studied. In contrast, the Nile tilapia (*O. niloticus*) and its traditional strains show relatively limited salinity tolerance. Therefore, despite being the most widely cultured and economically valuable species, the Nile tilapia has inherent limitations in salinity tolerance, making systematic research on its adaptation to saline environments particularly important for promoting industry expansion into salinity-fluctuating regions. As an important improved strain of Nile tilapia, the Genetically Improved Farmed Tilapia (GIFT) has demonstrated relatively good adaptability in brackish water aquaculture [14]. However, salinity stress still significantly affects osmotic balance, growth performance, and survival rate [15]. Thus, enhancing the salinity tolerance of this strain has become a key research priority. Current understanding of fish salinity tolerance has primarily emerged from studies on species such as rainbow trout (*Oncorhynchus mykiss*) [16] and Atlantic salmon (*Salmo salar*) [17]. In contrast, research on the molecular mechanisms underlying salinity adaptation in tilapia, particularly the GIFT strain, remains relatively limited. While some studies have analyzed gene expression or metabolic responses in gill tissues under salinity stress, revealing their direct functions in ion transport and osmoregulation, these investigations have largely focused on the immediate stress responses of localized tissues [14]. Currently, systematic integrated analyses combining transcriptomic and metabolomic approaches to study salinity adaptation in fish remain notably insufficient. Particularly regarding the liver—the central organ for energy metabolism and global regulation—the mechanisms by which it coordinates molecular changes across different biological levels in response to salinity stress remain unclear. This knowledge gap is especially critical given the systemic nature of stress adaptation. Therefore, this study focuses on liver tissue, aiming to systematically elucidate the molecular mechanisms underlying GIFT’s adaptation to salinity stress by identifying key regulatory pathways and metabolic shifts. To this end, we established controlled salinity gradients—freshwater and brackish water—and conducted integrated transcriptomic and metabolomic analyses on liver tissue. This strategy enables a comprehensive comparison of differentially expressed genes and significantly differential metabolites, with a focus on identifying molecular features associated with salinity tolerance. Based on KEGG pathway enrichment analysis, we further dissected the metabolic regulatory networks in the liver under salinity stress, thereby providing new insights into the understanding of environmental adaptation in teleosts from the perspective of energy allocation and metabolic remodeling, and establishing a scientific foundation for breeding salinity-resilient tilapia varieties.

## 2. Materials and Methods

### 2.1. Experimental Design and Sample Collection

The Nile tilapia GIFT strain (*Oreochromis niloticus*) used in this study was propagated at the Guangxi National Tilapia Breeding Farm. Ten broodstock fish (♀:♂ = 1:1) were randomly selected from the GIFT parental population for artificial insemination and incubation. The resulting progeny underwent 20 days of sex reversal followed by 30 days of intensive cultivation. A total of 240 healthy juvenile fish with uniform body size (25 ± 2 g) were selected and randomly allocated to two salinity treatment groups: the freshwater group and the brackish water group.

To ensure successful adaptation of the brackish water group to high-salinity conditions, a progressive salinity acclimation protocol was implemented prior to the formal experiment. All fish were first maintained in freshwater (0‰) for one week. Subsequently, the salinity for the brackish water group was gradually increased from freshwater to the target concentration of 24‰ at a daily rate of 3–4‰ by adding sea salt. (The composition of sea salt includes refined salt, magnesium chloride, calcium chloride, potassium chloride, etc.) This stepwise acclimation procedure was designed to minimize acute stress response caused by sudden salinity changes.

The brackish water group was reared in Qinzhou City, Guangxi Zhuang Autonomous Region (21.45° N, 108.34° E), where 120 fish were randomly distributed into three net cages (4 × 4 × 1.5 m each) with water salinity maintained at 24‰. The freshwater group was established in freshwater conditions in Qinzhou City, Guangxi Zhuang Autonomous Region (21.97° N, 108.51° E), where 120 fish were randomly distributed into three net cages of identical dimensions with water salinity maintained at 0‰. Each net cage contained 40 fish. All net cages were maintained in water with temperature ranging from 28 to 30 °C, pH between 7.0 and 8.0, and dissolved oxygen concentration greater than 5.0 mg/L. The fish were fed twice daily with formulated feed containing more than 32% crude protein. Any remaining feed was removed from the net cages within 1 h after each feeding to ensure feeding adequacy and maintain water quality. (The feed formulation is detailed in the Table S1.)

After a 2-month culture period, the tilapia were sampled for morphological measurements and dissection. The experiment was conducted in accordance with the guidelines of the Declaration of Helsinki and was approved by the FFRC Laboratory Animal Welfare and Ethics Committee. Experimental fish were anesthetized using MS-222 before dissection. Liver tissues (approximately 200 mg per fish) were collected, immediately snap-frozen in liquid nitrogen, and stored at −80 °C for subsequent transcriptomic and metabolomic analyses.

At the end of the two-month culture experiment, all experimental fish were anesthetized using MS-222 for morphological measurements. The following four key growth traits were recorded:

Body weight (BW): Measured using an electronic balance with an accuracy of 0.1 g.

Body length (BL): Measured as the straight-line distance from the snout tip to the end of the caudal peduncle.

Body depth (BWD): Measured as the vertical distance at the thickest part of the fish body, typically anterior to the origin of the dorsal fin.

All linear measurements were taken using digital calipers with an accuracy of 0.1 cm. Prior to statistical analysis, all traits were tested for normality and homogeneity of variances.

To evaluate the impact of salinity stress on the growth performance of GIFT, body weight (BW), body length (BL), and body depth (BWD) were measured after a two-month culture period in freshwater and brackish water (24‰). Prior to statistical analysis, data normality and homogeneity of variances were assessed. Based on these assumptions, unpaired *t*-tests were applied to variables meeting parametric requirements, whereas the non-parametric Mann–Whitney U test was used when these assumptions were not fully satisfied.

### 2.2. RNA Extraction, Library Construction, and Sequencing

For transcriptome analysis, each group had 4 biological replicates, with each replicate consisting of a pool of liver tissue from 4 individuals with consistent growth performance. Total RNA was extracted using TRIzol reagent (Invitrogen, Carlsbad, CA, USA). RNA integrity was checked by 1% agarose gel electrophoresis, and concentration/purity were measured using a NanoDrop Lite spectrophotometer (Thermo Fisher Scientific, Waltham, MA, USA). Libraries were constructed using the TruSeq RNA Library Prep Kit (Illumina, San Diego, CA, USA) (starting RNA amount 1 μg) and sequenced on the Illumina HiSeq 4000 platform (Illumina, San Diego, CA, USA).

### 2.3. Transcriptome Data Analysis

High-quality clean reads were obtained after filtering raw data (Q20 > 96%, Q30 > 93%, GC content 48%~51%). Transcriptome sequencing of tilapia liver tissue was performed, and the data acquired from the Illumina HiSeq 4000 platform were analyzed as follows: First, fastp (version 0.18.0) was used for quality control and filtering of raw reads to obtain high-quality clean reads; then, Trinity (version 2.8.4) software was used for de novo assembly to generate Unigene sequences, and assembly quality was assessed by BUSCO; blastx was used to align Unigenes to the NR, SwissProt, KEGG, and COG/KOG databases (E-value < 0.00001) for functional annotation; RSEM (v1.3.3) software was used to calculate gene expression levels (FPKM and TPM), and edgeR (version 1.20.0) was used to screen for differentially expressed genes (DEGs) with thresholds of FDR < 0.05 and |log_2_FC| > 1; finally, GO functional and KEGG pathway enrichment analyses were performed on the DEGs to reveal their biological functions and metabolic pathways.

### 2.4. Metabolome Analysis

Liver tissue metabolites were detected using liquid chromatography–electrospray tandem mass spectrometry (LC-ESI-MS/MS), combining both positive ion (POS) and negative ion (NEG) modes to improve metabolite coverage. After preprocessing, qualification, and quantification of the raw data, the analysis was performed following the referenced “Non-targeted Metabolomics Analysis Method” (MetWare Biotechnology Co., Ltd. Wuhan, China) as outlined below: First, data quality control was performed using QC samples to assess data stability. Subsequently, multivariate statistical analysis was conducted, including unsupervised Principal Component Analysis (PCA) and supervised Orthogonal Partial Least Squares-Discriminant Analysis (OPLS-DDA), with model reliability verified by permutation testing. Significantly differential metabolites (SDMs) were initially screened using the thresholds of Variable Importance in Projection (VIP) ≥ 1 and *t*-test *p* < 0.05. To further control the false positive rate, the *p*-values from the *t*-test were subjected to False Discovery Rate (FDR) correction, with the final screening criteria set as VIP ≥ 1 and FDR < 0.05. SDMs were then analyzed using volcano plots, cluster heatmaps, and Z-score analysis. Finally, KEGG database was used for pathway annotation of the differential metabolites, and hypergeometric test was applied for pathway enrichment analysis (FDR ≤ 0.05) to reveal the significantly altered metabolic pathways in tilapia liver under salinity stress.

### 2.5. Transcriptome and Metabolome Integrated Analysis

The KEGG pathway annotation results of DEGs and SDMs were integrated to identify co-enriched pathways and construct gene–metabolite interaction networks.

## 3. Results

### 3.1. Growth Performance in Response to Salinity Stress

**Figure 1:**
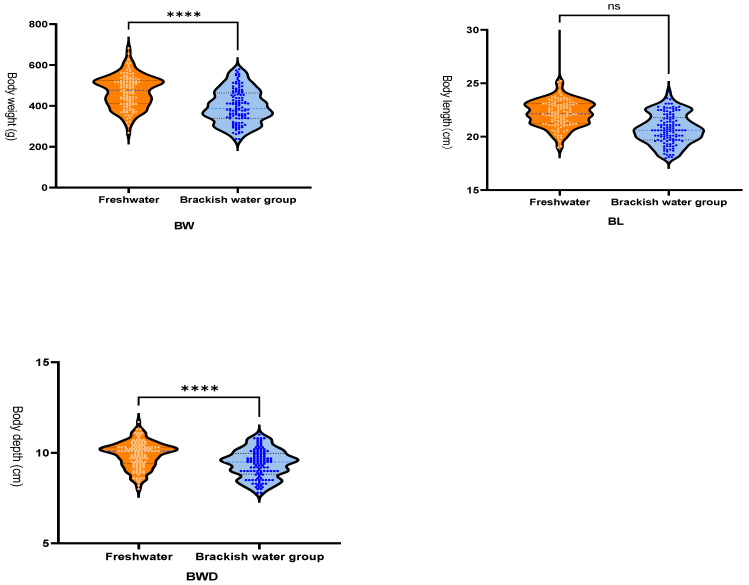
Growth Data. Body Weight (BW). Body Length (BL). Body Depth (BWD). Freshwater group growth rate: 1791% (daily weight gain: 7.46 g/day); Brackish water group growth rate: 1488% (daily weight gain: 6.20 g/day). The blue dots represent individuals from the brackish water group, and the red dots represent individuals from the freshwater group. “****” indicates a highly significant difference (*p* < 0.0001), and “ns” indicates no significant difference (*p* > 0.05).

As shown in Figure 1, salinity stress induced a highly significant reduction in body weight (BW, *p* < 0.0001) and body depth (BWD, *p* < 0.0001). In contrast, the decrease in body length (BL) did not reach statistical significance (*p* = 0.061, ns). These results demonstrate that salinity stress exerts distinct effects on different morphological traits of GIFT, with pronounced inhibition of weight and depth, a strong alteration in width, but no significant impact on length under the present experimental conditions.

### 3.2. Quality Assessment of Sequencing Data

In the transcriptome sequencing of tilapia liver, the raw reads from the 8 libraries ranged from 36,185,740 to 52,918,546. The proportion of clean reads was 99.40%~99.68%, Q20 > 96%, Q30 > 93%, and GC content 48%~51%. The sequencing quality was high, meeting the requirements for subsequent analysis (Detailed data are provided in the Supplementary Materials).

### 3.3. Identification of DEGs

**Figure 2:**
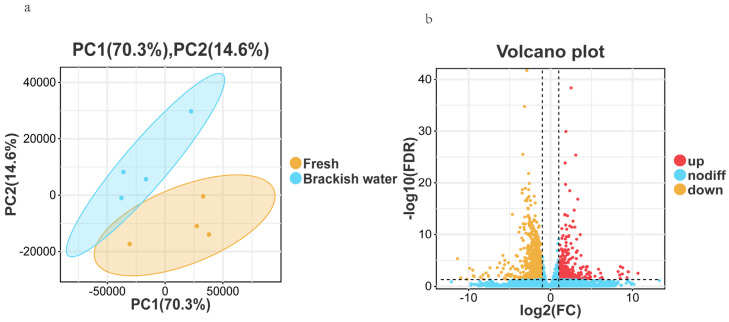
(**a**): Principal Component Analysis (PCA) plot; (**b**): Volcano plot of differentially expressed genes.

Principal Component Analysis (PCA) results showed clear separation between the Fresh (control group) and Brackish water group (experimental group) samples in the PCA plot (Figure 2a), with PC1 contributing 70.3% and PC2 contributing 14.6% of the variance. This indicates that the gene expression patterns of the two groups were significantly different due to salinity stress, with good intra-group sample clustering, and inter-group differences being the main factor affecting gene expression. Principal Component Analysis (PCA) results showed clear separation between the Fresh and Brackish water group samples in the PCA plot (Figure 2a), with PC1 contributing 70.3% and PC2 contributing 14.6% of the variance. This indicates that the gene expression patterns of the two groups were significantly different due to salinity stress, with good intra-group sample clustering, and inter-group differences being the main factor affecting gene expression. Using thresholds of FDR < 0.05 and |log_2_FC| > 1 for differential analysis, a total of 1529 differentially expressed genes (DEGs) were identified. Compared with the freshwater group, 399 genes were significantly up-regulated and 1130 genes were significantly down-regulated in the brackish water group (Figure 2b).

### 3.4. Enrichment Analysis of DEGs

**Figure 3:**
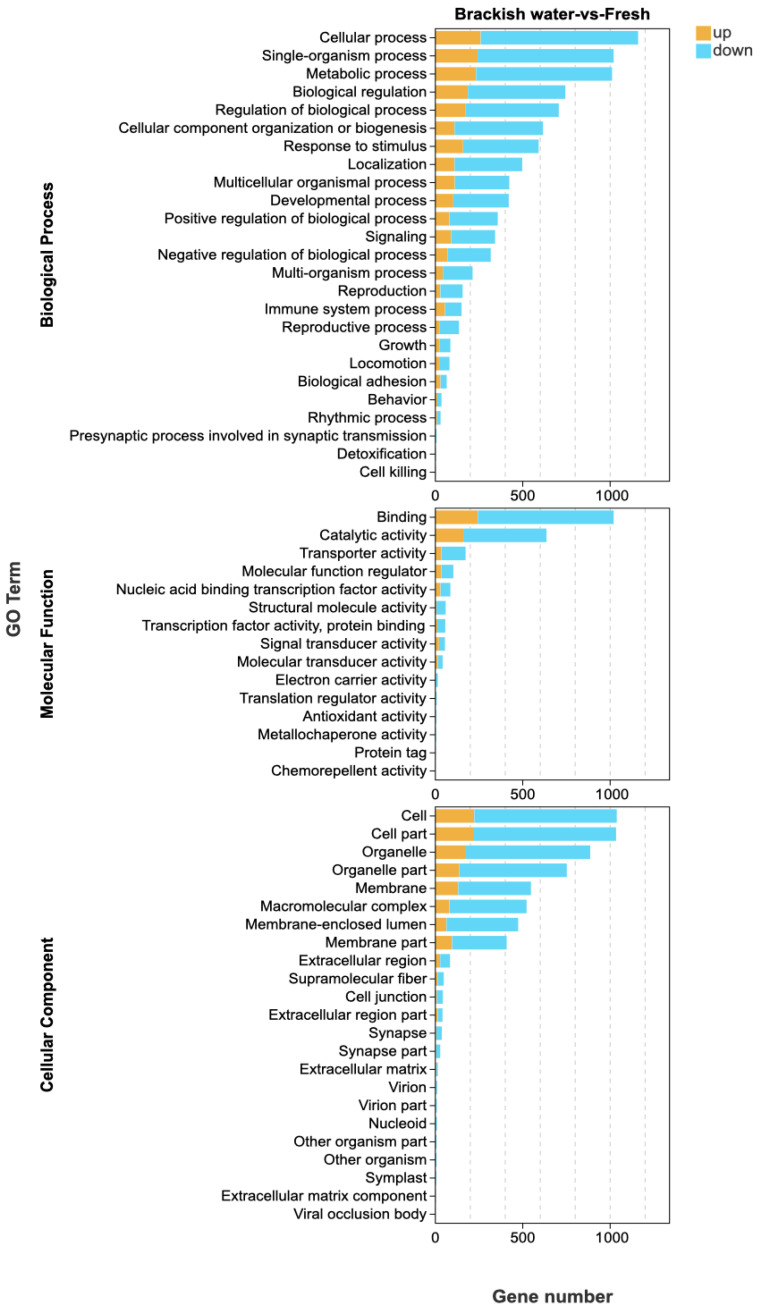
GO enrichment analysis of differentially expressed genes in tilapia liver under salinity stress. This figure displays the significantly enriched GO terms (FDR < 0.05) for genes up-regulated in the brackish water group compared to the freshwater group, categorized into Biological Process, Cellular Component, and Molecular Function. The x-axis represents the number of annotated genes. Only the top 25 most enriched terms in the Biological Process category are shown.

**Figure 4:**
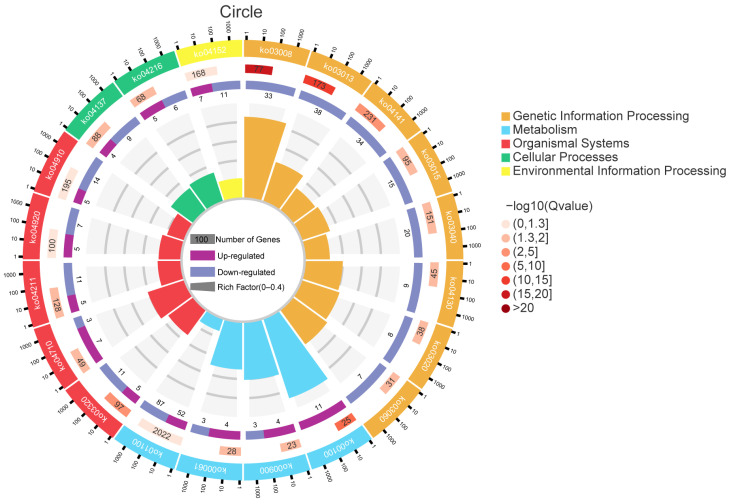
KEGG enrichment analysis of differentially expressed genes in tilapia liver under salinity stress. The figure displays the most significantly enriched KEGG pathways. Each dot represents a pathway, its position indicates the enrichment factor (Rich Factor, number of DEGs mapped to the pathway/number of all annotated genes mapped to the pathway) and enrichment significance (−log_10_(FDR)). The dot size corresponds to the number of DEGs mapped to the pathway, and the color corresponds to different pathway categories. KEGG pathway enrichment analysis further revealed that DEGs were significantly enriched in multiple metabolic pathways, among which “Steroid biosynthesis”, “Glycolysis/Gluconeogenesis”, and “Fatty acid biosynthesis” were prominent.

**Figure 5:**
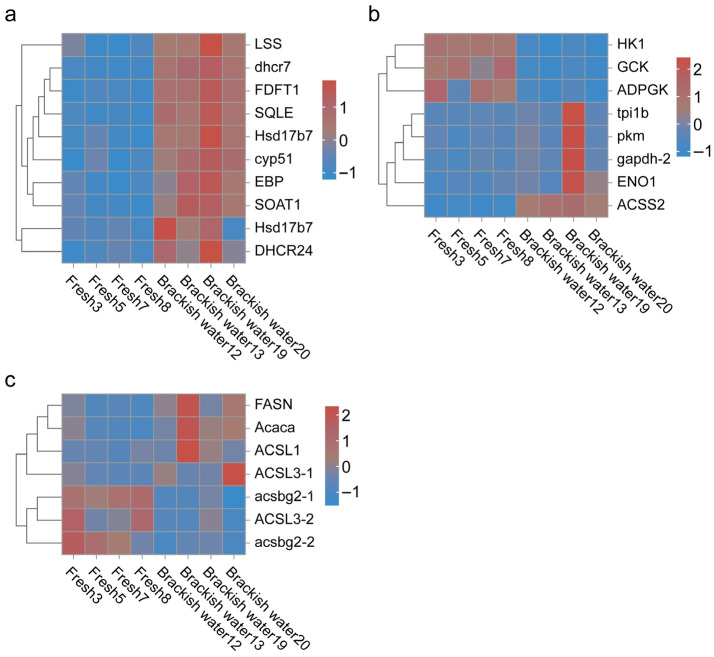
Impact of salinity stress on key metabolic pathways in tilapia. (**a**): Heatmap of DEGs in Steroid biosynthesis; (**b**): heatmap of DEGs in Glycolysis/Gluconeogenesis; (**c**): heatmap of DEGs in Fatty acid biosynthesis. The expression heatmaps of genes in these pathways (**a**–**c**) clearly show the differential patterns between groups, indicating that salinity stress severely disrupted lipid metabolism and energy metabolism homeostasis in tilapia. Each row represents a gene, and each column represents a sample. Red indicates high expression levels, blue indicates low expression levels.

GO functional enrichment analysis showed that a total of 63 terms were enriched by the 1529 differentially expressed genes (DEGs) identified in the brackish water group (experimental group) compared with the freshwater group (control group) (Figure 3). In the Biological Process (BP) category, ‘cellular process’ and ‘single-organism process’ were the main terms, involving basic life activities such as cell proliferation and substance metabolism, suggesting that salinity stress may regulate the adaptation mechanisms of tilapia by affecting basic cellular functions. In the Cellular Component (CC) category, ‘cell’ and ‘cell part’ had the highest proportions, indicating that salinity stress might affect the integrity of cellular structures. In the Molecular Function (MF) category, the terms ‘binding’ and ‘catalytic activity’ were significantly enriched, involving processes such as enzymatic reactions and signal molecule binding, implying that the tilapia liver might cope with environmental changes by regulating protein binding and catalytic functions under salinity stress. KEGG pathway enrichment analysis (Figure 4) further revealed that DEGs were significantly enriched in multiple metabolic pathways, among which “Steroid biosynthesis”, “Glycolysis/Gluconeogenesis”, and “Fatty acid biosynthesis” were prominent. Under salinity stress, the expression of genes in the steroid biosynthesis pathway was significantly up-regulated, indicating that the organism may activate steroid hormone synthesis pathways to cope with osmoregulatory or stress-related demands. In the glycolysis/gluconeogenesis pathway, the mixed pattern of gene up-regulation and down-regulation indicated that salinity stress triggered a reprogramming of energy metabolism pathways, with fish likely adjusting their carbohydrate utilization and production strategies to meet the energy demands of osmoregulation. In the fatty acid biosynthesis pathway, gene expression exhibited a differential pattern. Among them, acsbg2 showed consistent down-regulation across all biological replicates in the brackish water group. ACSL3 also displayed a down-regulation trend, though its extent varied among replicate samples. In contrast, other key genes such as FASN, Acaca, and ACSL1 showed mixed expression patterns across replicates, without forming a consistent down-regulation trend (Figure 5).

### 3.5. Identification of SDMs

**Figure 6:**
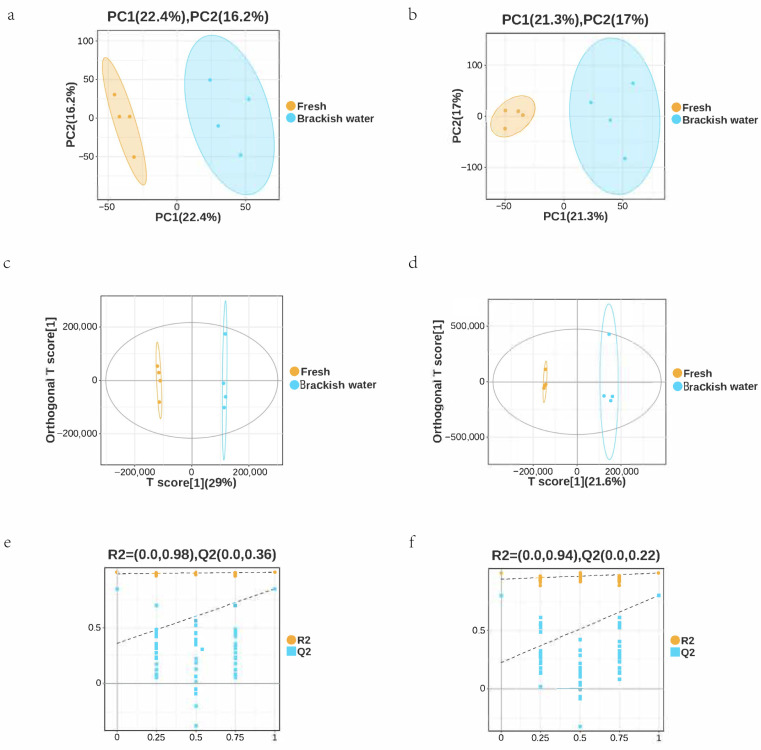
(**a**) PCA score plot of the freshwater (pos) group. (**b**) PCA score plot of the brackish water (neg) group. (**c**) PLS-DA score plot of the freshwater group. (**d**) PLS-DA score plot of the brackish water group. (**e**) Model validation plot for the freshwater group. (**f**) Model validation plot for the brackish water group. The percentages in parentheses in plots (**a**–**d**) indicate the variance explained by each component. The R2 and Q2 values in (**e**,**f**) represent the goodness of fit and predictive ability of the model, respectively, obtained from cross-validation.

Metabolome analysis of tilapia liver detected a total of 871 metabolites. PCA results showed clear separation between the Fresh and Brackish water groups in both positive and negative ion modes. In positive ion mode, PC1 contributed 22.4% and PC2 contributed 16.2% (Figure 6a); in negative ion mode, PC1 was 21.3% and PC2 was 17% (Figure 6b). Intra-group samples clustered tightly, indicating that salinity stress significantly altered the liver metabolic profile. The OPLS-DA model validation showed good model stability, effectively distinguishing the metabolic differences between the two groups (Figure 6c–f).

**Figure 7:**
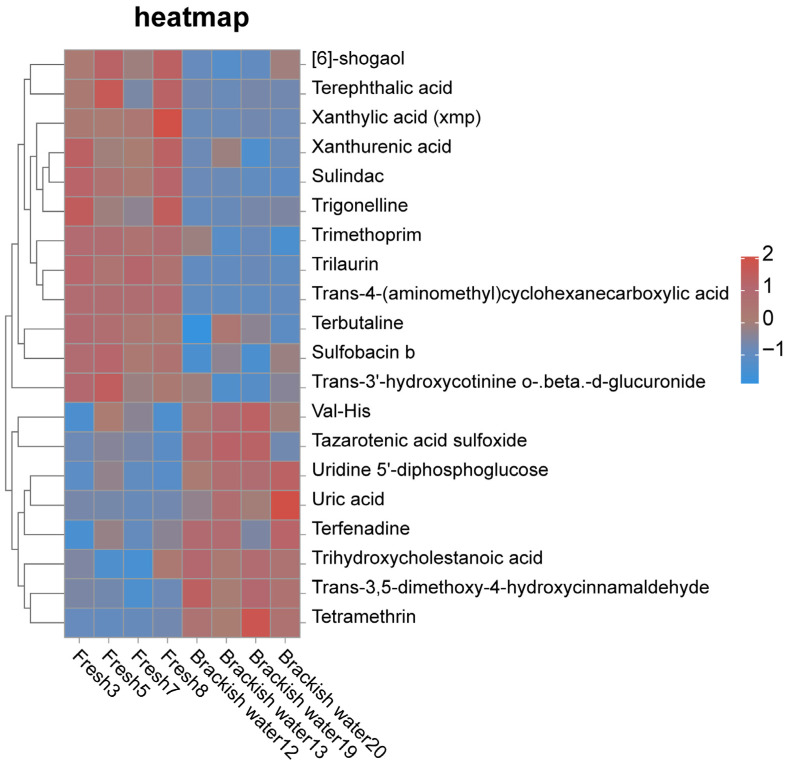
Cluster heatmap of significantly different metabolites between Fresh and Brackish water groups.

The cluster heatmap (Figure 7) showed distinct clustering characteristics of significantly differential metabolites (SDMs) between the two groups. Compared with the brackish water group, the levels of metabolites such as Terephthalic acid, Xanthurenic acid, and Trilaurin were significantly higher in the freshwater group. Conversely, nearly 50% of the identified SDMs, including metabolites such as Val-His and Uridine 5′-diphosphoglucose, exhibited the opposite pattern, with significantly lower levels in the freshwater group.

### 3.6. Enrichment Analysis of SDMs

**Figure 8:**
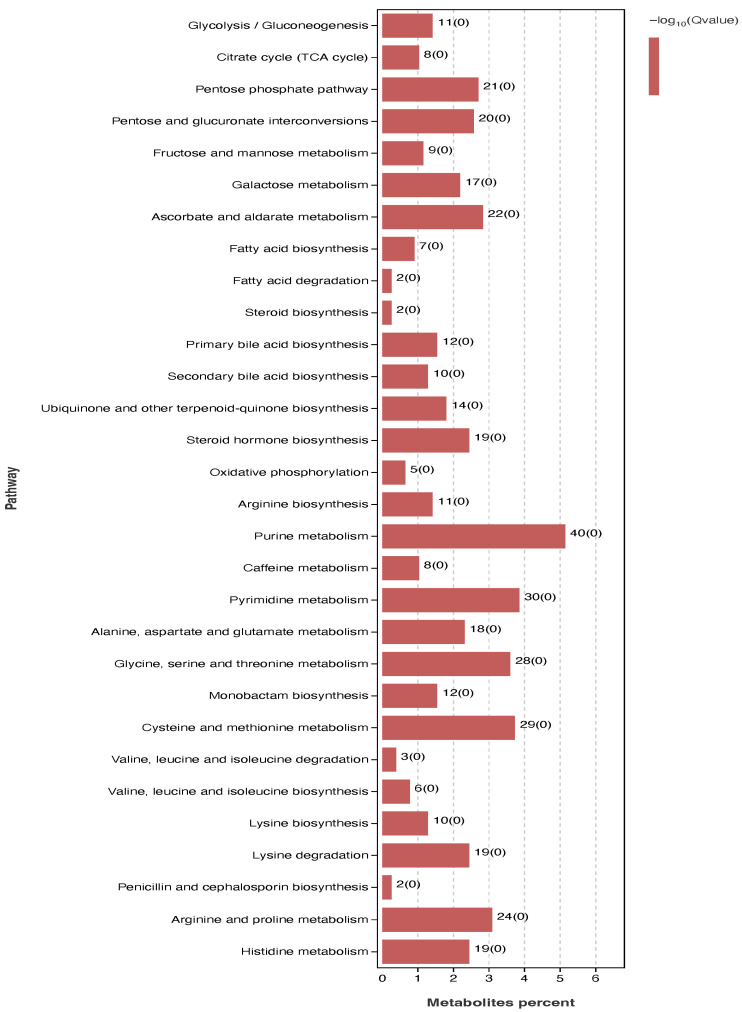
Bar plot of KEGG pathway enrichment analysis for significantly different metabolites in tilapia liver.

KEGG pathway enrichment analysis revealed that the 127 significantly differential metabolites (SDMs) were annotated to 30 metabolic pathways (Figure 8). The most prominently enriched pathways included the pentose phosphate pathway, pentose and glucuronate interconversions, steroid hormone biosynthesis, and fatty acid biosynthesis. The significance level (−log_10_(FDR)) and enrichment factor (Rich factor) for each pathway are detailed in Figure 8.

### 3.7. Integrated Analysis of Transcriptomics and Metabolomics Data

Integrated analysis revealed that 30 pathways were annotated with both DEGs and SDMs, including the Chemokine signaling pathway involved in immune response, metabolic pathways related to nucleotide metabolism, and cell signaling pathways such as PI3K-Akt and MAPK. Co-enrichment analysis showed significant enrichment in pathways like the AMPK signaling pathway and Amino acid biosynthesis, which are related to energy metabolism and neural signal transduction.

**Figure 9:**
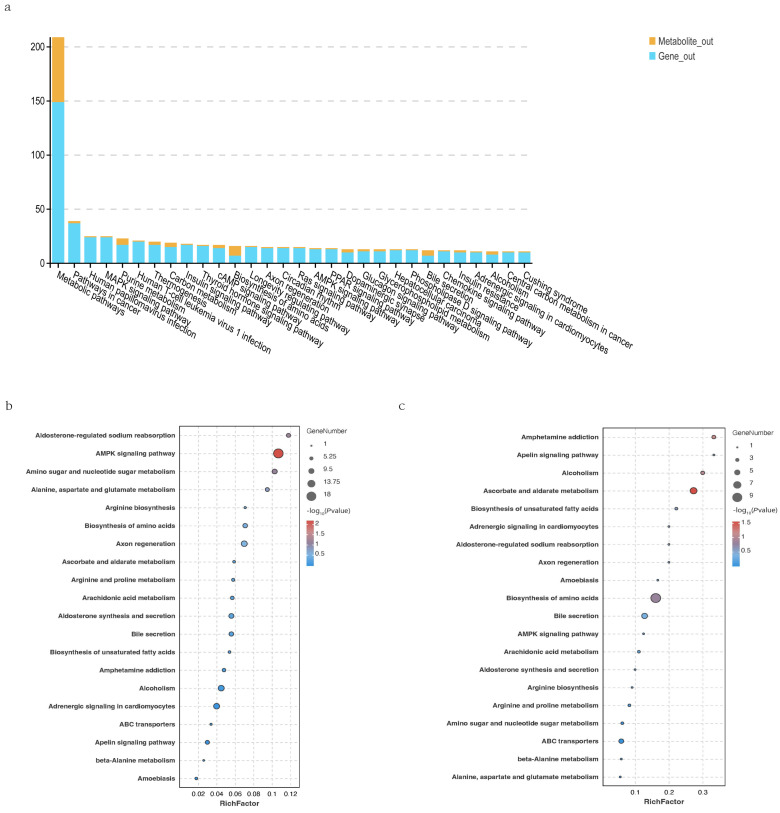
(**a**): Pathways commonly associated with differentially expressed genes and significantly different metabolites between Fresh and Brackish water groups. (**b**): Transcriptome KEGG enrichment analysis of differentially expressed genes. (**c**): Metabolome KEGG enrichment analysis of differential metabolites.

As shown in Figure 9, KEGG enrichment analysis of DEGs and SDMs showed significant enrichment in pathways such as “AMPK signaling pathway”, “Ascorbate and aldarate metabolism”, “Biosynthesis of amino acids”, “Apelin signaling pathway”, and “Amphetamine addiction”. These pathways are related to energy metabolism and neural signal transduction.

## 4. Discussion

Soil and water salinization pose significant threats to global agricultural productivity [18]. Driven by a combination of natural factors, including climate warming [19], increased evaporation, and shifting precipitation patterns [20], as well as anthropogenic activities, the extent of saline-alkaline waters worldwide is increasing annually [21]. Consequently, the sustainable and comprehensive utilization of brackish water resources has become an issue of considerable ecological and economic importance. As a key environmental factor, salinity directly modulates a wide range of physiological processes in aquatic animals. While studies have shown that optimal salinity levels can enhance feed conversion efficiency, feeding rates, growth performance, and muscle quality in fish, excessively high salinity induces oxidative stress and inflicts damage on tissues such as the liver.

Research on salinity stress in aquatic species has advanced considerably, now routinely incorporating analyses at physiological, biochemical, and molecular levels. For instance, the common carp responds to salinity stress primarily through hepatic lipid metabolism dysregulation and the ferroptosis pathway [22]. In contrast, stenohaline fish such as common carp (*Cyprinus carpio*) [23] may exhibit more pronounced metabolic dysregulation and antioxidant system activation under salinity stress [24]. Although both involve oxidative stress and metabolic regulation, their central pathways and primary organs of response differ significantly. In contrast, the GIFT (Oreochromis niloticus), a euryhaline farmed fish species, possesses inherent salt tolerance, yet a systematic molecular-level understanding of its response mechanisms to fluctuating salinity remains largely unexplored.

In this study, we employed a comparative transcriptomic and metabolomic analysis of the liver in GIFT reared in freshwater and brackish water environments. Our findings demonstrate that salinity stress significantly disrupts metabolic homeostasis. KEGG enrichment analysis identified Steroid biosynthesis, Glycolysis/Gluconeogenesis, and Fatty acid biosynthesis as the most significantly altered metabolic pathways in response to salinity change. These pathway alterations collectively reflect the organism’s adaptive strategies concerning osmoregulation, energy supply, and membrane structure remodeling. In this study, the overall decline in the growth performance of GIFT tilapia in brackish water (24‰) (Figure 1) may be related to the reallocation of energy by the organism in response to osmotic pressure changes. It is important to clarify that the physiological challenges and energy demands faced by fish in freshwater (undergoing hypo-osmoregulation) and brackish water (undergoing hyper-osmoregulation) are fundamentally different [25]. The metabolic remodeling observed—particularly the inhibition of anabolic processes such as steroid and fatty acid synthesis, along with the reprogramming of energy metabolism pathways—may indicate that when fish transition from a hypo-osmotic to a hyper-osmotic environment, resources are prioritized to maintain ionic balance and osmotic homeostasis. While this adaptive strategy aids short-term survival, it also limits investment in growth. Future research could further quantify the efficiency of energy allocation under different osmoregulatory modes and its specific impact on growth by integrating energy budget measurements with multiple physiological indicators. The metabolomic results provide further evidence for this metabolic remodeling at the substance level. We observed that the levels of metabolites with potential antioxidant functions, such as terephthalic acid and xanthurenic acid, were higher in the liver of the freshwater group, which may contribute to maintaining a lower oxidative stress state. Furthermore, the significant alterations in lipid metabolites, such as trilaurin, echo the down-regulation of the fatty acid biosynthesis pathway revealed by transcriptomics, jointly pointing to profound changes in hepatic lipid storage and turnover. However, in this study, we focused exclusively on liver tissue, whereas salinity adaptation involves multi-organ coordination; only a single salinity level (24‰) was tested, which prevents the analysis of dose-dependent effects; and aside from growth indicators, key physiological parameters such as osmoregulatory capacity were not measured, limiting the functional interpretation of the omics data. Future research that integrates multi-tissue analysis, gradient salinity designs, and physiological parameter validation will help construct a more comprehensive systemic model of tilapia salinity adaptation, thereby providing a more solid scientific foundation for breeding salt-tolerant varieties and guiding aquaculture practices.

Steroid Biosynthesis Pathway in GIFT

Steroid biosynthesis is a recognized component of animal lipid metabolism [26] and plays a role in aquatic animals’ responses to environmental stressors such as salinity changes [27]. This pathway initiates from acetyl-CoA and constitutes a crucial metabolic process involving multiple enzymatic steps to produce cholesterol and other steroid hormones [28]. Sterols serve not only as essential components of cell membranes but also as precursors for steroid hormones that regulate osmotic balance and stress responses, underscoring the pathway’s significance in fish salinity adaptation. Our transcriptomic data revealed a significant up-regulation of most genes within the steroid biosynthesis pathway in the brackish water group (Figure 5a). This finding suggests that salinity stress may activate the transcriptional activity of this pathway to meet the increased hormonal demands associated with osmoregulation or stress responses. Steroid hormones such as cortisol are key regulators of environmental stress responses in fish [29]. Typically, cortisol levels rise during the initial phase of salinity stress to activate ion transport systems. The up-regulated gene expression pattern observed in this study aligns with this physiological response mechanism, suggesting that the organism may enhance steroid hormone synthesis capacity to cope with osmoregulatory challenges under salinity stress. This transcriptional change is further supported at the metabolite level, as significantly altered metabolites are enriched in the “Steroid hormone biosynthesis” pathway (Figure 8). However, under prolonged or intense stress conditions, the regulation of steroid synthesis in the organism may become more complex. Although transcriptional activity is enhanced, the final efficiency of steroid hormone synthesis may be influenced by multiple factors such as post-translational regulation, enzyme activity, substrate availability, or hormone turnover rates [30]. It is noteworthy that steroid hormone synthesis heavily depends on the supply of precursors such as acetyl-CoA. Therefore, salinity stress may indirectly regulate the biosynthetic flux of hormones by affecting upstream lipid metabolism and energy homeostasis [6]. Furthermore, since steroid hormones are also involved in regulating antioxidant and immune responses [31], their dynamic changes in synthesis during salinity adaptation may be closely linked to the overall stress adaptation capacity of fish. The up-regulation of the steroid biosynthesis pathway observed in this study, combined with perturbations at the metabolite level, reflects the active involvement of this pathway in salinity adaptation. However, its specific functional output still requires further validation through direct measurement of hormone levels.

Functionally, steroid hormones are categorized into corticosteroids and sex hormones. Corticosteroids promote gluconeogenesis and protein metabolism, whereas sex hormones play a role in stimulating protein synthesis, particularly in muscle and reproductive organ development [32]. Sex hormones may also influence fish growth by modulating growth hormone (GH) secretion and mediating the GH-IGF1 signaling axis [33]. Therefore, the activation of the steroid biosynthesis pathway under salinity stress may regulate the growth performance, metabolic balance, and stress adaptation of GIFT by affecting these interconnected hormonal regulatory networks.

Fatty Acid Biosynthesis Pathway in GIFT

Fatty acids, which are universally indispensable biomolecules across all living organisms [34], serve as fundamental constituents of fats, phospholipids, and glycolipids. They play critical roles as structural components of cell membranes and as a primary energy source [35]. Its biosynthesis begins with acetyl-CoA [36] and proceeds through multiple steps catalyzed [37] by key enzymes such as acetyl-CoA carboxylase (Acc) [38] and fatty acid synthase (Fasn) [39]. In the saline water group, the expression of key genes involved in de novo fatty acid synthesis, such as Fasn, and genes responsible for fatty acid activation, such as long-chain acyl-CoA synthetases (e.g., ACSL1 and ACSL3), was significantly upregulated. This indicates that under high-salinity stress, the liver simultaneously enhances both de novo fatty acid synthesis and the activation of existing fatty acids, resulting in a coordinated metabolic mobilization.

The synchronous upregulation of Fasn and ACSL genes indicates that the organism does not simply regulate individual metabolic pathways in a unidirectional manner but rather initiates systemic metabolic reprogramming. This coordinated response likely aims to rapidly enhance intracellular lipid reserves, allowing newly synthesized fatty acids to be efficiently activated and directed toward processes such as energy storage and membrane system remodeling.

Taken together, these findings suggest that salinity stress induces a “lipid mobilization” response through the concurrent upregulation of genes involved in fatty acid synthesis and activation. This metabolic shift reallocates resources toward building lipid reserves and maintaining membrane integrity—a strategy that supports short-term adaptation to saline conditions, though potentially at the expense of metabolic investment in growth-related processes.

Glycolysis/Gluconeogenesis

Glycolysis and gluconeogenesis are core, antagonistic pathways in glucose metabolism, working in concert to maintain blood glucose homeostasis and energy supply under varying physiological conditions. Glycolysis, occurring in the cytoplasm, breaks down glucose to pyruvate with a net yield of 2 ATP, serving as a critical pathway for rapid energy generation during hypoxia or high-intensity exercise [40]. Specifically, glycolysis is the process in which glucose is broken down into pyruvate. Under anaerobic conditions, pyruvate is further metabolized into lactate or ethanol, producing 2 molecules of ATP. This process serves as a primary energy acquisition pathway for organisms under anaerobic conditions, such as during intense exercise [41,42]. In contrast, gluconeogenesis occurs predominantly in the liver and synthesizes glucose from non-carbohydrate precursors like lactate, amino acids, and glycerol, playing a vital role in maintaining blood glucose levels during starvation or stress [43]. Of particular note is the discovery that gluconeogenesis is subject to transcriptional regulation in fish [44,45].

The significant enrichment of the “Glycolysis/Gluconeogenesis” pathway in our KEGG analysis (Figure 4) indicates its central role in the hepatic response of GIFT to salinity stress. Our transcriptomic data revealed (Figure 5b) that the expression of multiple key genes was significantly altered. Among these, genes encoding hexokinase (hk1, gck) and pyruvate kinase (pkm) exhibited an overall downregulation trend in the brackish water group. These enzymes are key rate-limiting steps in glycolysis [46,47], and their downregulation strongly suggests that hepatic glycolytic flux may be suppressed under prolonged salinity stress. Concurrently, the upregulation of some genes within the pathway may reflect compensatory adjustments at specific nodes or a redirection of energy demands.

We propose that this complex pattern of gene expression changes reflects the organism’s active, strategic reallocation of energy and metabolic intermediates in response to salinity stress. Salinity stress imposes a high energetic demand. By inhibiting key glycolytic steps, the organism may reduce the net ATP output from glucose catabolism, thereby conserving energy substrates such as glucose and ATP, and prioritizing essential functions like osmoregulation. Our integrated multi-omics analysis (Figure 9) further identified the “Glycolysis/Gluconeogenesis” pathway as a key hub highlighted by both transcriptomic and metabolomic data, indicating that changes in this pathway occur not only at the transcriptional level but also lead to significant alterations at the metabolic level. This fine-tuning of central carbon metabolism, along with the differential expression patterns observed in pathways such as steroid biosynthesis (Figure 5a), collectively suggests that the organism does not simply suppress metabolism across the board but undergoes targeted and selective reprogramming.

In summary, the remodeling of gene expression in the glycolysis/gluconeogenesis pathway constitutes a critical component of the systemic metabolic adaptation of GIFT to salinity stress. The underlying regulatory logic involves modulating the direction and intensity of central carbon metabolism to conserve energy, support osmoregulation, and simultaneously provide necessary precursors for other adaptive anabolic processes. Notably, changes in the levels of metabolites such as uridine 5′-diphosphoglucose and xanthylic acid in the metabolomic data further support the adaptive adjustments in glycosylation reactions and energy metabolism at the metabolite level. This resource reallocation strategy, which shifts resources from basal growth metabolism toward stress response, enhances short-term adaptability but inevitably reduces the resources available for growth.

## 5. Conclusions

This study systematically elucidated the metabolic remodeling mechanisms in the liver of GIFT under salinity stress through integrated transcriptomic and metabolomic analyses, identifying a total of 1529 differentially expressed genes and 127 significantly differential metabolites. These molecules were predominantly enriched in three core pathways: steroid biosynthesis, glycolysis/gluconeogenesis, and fatty acid biosynthesis. The results indicate that the organism employs an adaptive strategy centered on energy reallocation by coordinated suppression of key anabolic pathways (such as steroid and fatty acid synthesis) and fine-tuned regulation of carbohydrate metabolism. This strategy prioritizes the allocation of resources to survival-maintaining functions such as osmoregulation, thereby providing a molecular-level explanation for the observed decline in growth performance. Further integrated multi-omics analysis revealed that this systemic metabolic adaptation is likely orchestrated by a central regulatory network: the significant activation of the AMPK signaling pathway and the reprogramming of amino acid metabolism together constitute a coordinated response mechanism to energy stress. These findings offer multiple insights for tilapia aquaculture in brackish water environments. First, they reveal the metabolic-level adaptive strategy of “survival over growth” in fish under salt stress. Second, understanding the mechanism of energy reallocation can guide the development of targeted nutritional regulation strategies. Additionally, the research results emphasize the importance of implementing gradual salinity acclimation in practical farming practices. Finally, the key genes and metabolites identified in the study can serve as candidate pathways for the selective breeding of salinity-tolerant traits.

## Data Availability

The data presented in this study are openly available. Raw sequence reads are deposited in the CNCB (accession number CRA033631).

## Supplementary Materials

The following supporting information can be downloaded at: https://www.mdpi.com/article/10.3390/vetsci13010105/s1, Table S1: Feed formulation; Table S2: Sequencing data.

## References

[1] Chen J.-C., Chen W.-C. (2000). Salinity Tolerance of *Haliotis diversicolor supertexta* at Different Salinity and Temperature Levels. Aquaculture.

[2] Lushchak V.I. (2011). Environmentally Induced Oxidative Stress in Aquatic Animals. Aquat. Toxicol..

[3] Dubey S.K., Trivedi R.K., Chand B.K., Mandal B., Rout S.K. (2017). Farmers’ Perceptions of Climate Change, Impacts on Freshwater Aquaculture and Adaptation Strategies in Climatic Change Hotspots: A Case of the Indian Sundarban Delta. Environ. Dev..

[4] Mohammadi M., Sarsangi Aliabad H., Shekarabi S.P.H., Ghaedi A., Alizadeh M., Nabi A., Bahmani M., Gharaei A., Akhavan-Bahabadi M. (2025). Reproductive Traits in Different Genetically Improved Nile Tilapia (*Oreochromis niloticus*) Strains Raised in Brackish Water. Aquac. Res..

[5] Akbar M.A., Jayanthi S., Fajri S., Zahara A.S., Sari M.T. (2023). The Influence of Different Media on The Physiological Response of Nila Fish (*Oreochormis nilaticus*). J. Biol. Trop..

[6] Zhu Z.X., Jiang D.L., Li B.J., Qin H., Meng Z.N., Lin H.R., Xia J.H. (2019). Differential Transcriptomic and Metabolomic Responses in the Liver of Nile Tilapia (*Oreochromis niloticus*) Exposed to Acute Ammonia. Mar. Biotechnol..

[7] Zhu J., Zou Z., Li D., Xiao W., Yu J., Chen B., Yang H. (2023). Comparative Transcriptomes Reveal Different Tolerance Mechanisms to Streptococcus Agalactiae in Hybrid Tilapia, Nile Tilapia, and Blue Tilapia. Fish Shellfish Immunol..

[8] Sunarto A., Grimm J., McColl K.A., Ariel E., Krishnankutty Nair K., Corbeil S., Hardaker T., Tizard M., Strive T., Holmes B. (2022). Bioprospecting for Biological Control Agents for Invasive Tilapia in Australia. Biol. Control.

[9] Haetami K., Harmonis J.A.A., Putri N.J.A., Nusyaibah K.A., Putri N.C., Syahputra M.R., Aulia R.F., Kusmana N.R. (2025). Mini Review Protein Value and The Importance of Energy Ratio (Case Study on *Oreochromis niloticus* Diet). J. Biol. Trop..

[10] Ashouri G., Hoseinifar S.H., El-Haroun E., Imperatore R., Paolucci M., Hoseinifar S.H., Van Doan H. (2023). Tilapia Fish for Future Sustainable Aquaculture. Novel Approaches Toward Sustainable Tilapia Aquaculture.

[11] Suresh A.V., Lin C.K. (1992). Tilapia Culture in Saline Waters: A Review. Aquaculture.

[12] Elangovan P., Ahilan B., Jeevagan I., Renuhadevi M. (2019). Tilapia—An Excellent Candidate Species for World Aquaculture: A Review. Annu. Res. Rev. Biol..

[13] Moorman B.P., Inokuchi M., Yamaguchi Y., Lerner D.T., Grau E.G., Seale A.P. (2014). The Osmoregulatory Effects of Rearing Mozambique Tilapia in a Tidally Changing Salinity. Gen. Comp. Endocrinol..

[14] Qin H., Yu Z., Zhu Z., Lin Y., Xia J., Jia Y. (2022). The Integrated Analyses of Metabolomics and Transcriptomics in Gill of GIFT Tilapia in Response to Long Term Salinity Challenge. Aquac. Fish..

[15] Li Y., Gao P., Zhou K., Yao Z., Sun Z., Qin H., Lai Q. (2024). Effects of Saline and Alkaline Stresses on the Survival, Growth, and Physiological Responses in Juvenile Mandarin Fish (*Siniperca chuatsi*). Aquaculture.

[16] Le Bras Y., Dechamp N., Krieg F., Filangi O., Guyomard R., Boussaha M., Bovenhuis H., Pottinger T.G., Prunet P., Le Roy P. (2011). Detection of QTL with Effects on Osmoregulation Capacities in the Rainbow Trout (*Oncorhynchus mykiss*). BMC Genet..

[17] Su Y., Yu S.-E., Sun Y.-X., Zhang L., Tan Y., Zhang Y.-Y., Wang S., Zhou Y.-G., Hu L.-S., Dong Y.-W. (2024). Genome-Wide Identification and Quantification of Salinity-Responsive Na^+^/K^+^-ATPase α-Subunits in Three Salmonids. Aquaculture.

[18] Tarolli P., Luo J., Park E., Barcaccia G., Masin R. (2024). Soil Salinization in Agriculture: Mitigation and Adaptation Strategies Combining Nature-Based Solutions and Bioengineering. iScience.

[19] Mukhopadhyay R., Sarkar B., Jat H.S., Sharma P.C., Bolan N.S. (2021). Soil Salinity under Climate Change: Challenges for Sustainable Agriculture and Food Security. J. Environ. Manag..

[20] Chen X., Liu X., Peng W., Dong F., Chen Q., Sun Y., Wang R. (2019). Hydroclimatic Influence on the Salinity and Water Volume of a Plateau Lake in Southwest China. Sci. Total Environ..

[21] Williams W.D. (2001). Anthropogenic Salinisation of Inland Waters. Hydrobiologia.

[22] Shang X., Che X., Geng L., Zhang Q., Wei H., Li W., Shi X., Xu W. (2025). Transcriptomic Analysis Reveals That Selenium-Enriched *Lactobacillus plantarum* Alleviates High-Salinity Stress in Common Carp through Lipid Metabolism and Ferroptosis Signalling Pathways. Ecotoxicol. Environ. Saf..

[23] Chen X., Li B., Hou Y., Wei K., Zhou L., Zhang C., Zhang L., Zhu J., Jia R. (2025). Physiological Responses and Serum Metabolite Alterations in Grass Carp (*Ctenopharyngodon idellus*) Under Chronic Salinity Exposure. Antioxidants.

[24] Djiba P.K., Zhang J., Xu Y., Zhang P., Zhou J., Zhang Y., Luo Y. (2021). Correlation between Metabolic Rate and Salinity Tolerance and Metabolic Response to Salinity in Grass Carp (*Ctenopharyngodon idella*). Animals.

[25] Bœuf G., Payan P. (2001). How Should Salinity Influence Fish Growth?. Comp. Biochem. Physiol. Part C Toxicol. Pharmacol..

[26] Si Y., Wen H., Li Y., He F., Li J., Li S., He H. (2018). Liver Transcriptome Analysis Reveals Extensive Transcriptional Plasticity during Acclimation to Low Salinity in Cynoglossus Semilaevis. BMC Genom..

[27] Xu Z., Gan L., Li T., Xu C., Chen K., Wang X., Qin J.G., Chen L., Li E. (2015). Transcriptome Profiling and Molecular Pathway Analysis of Genes in Association with Salinity Adaptation in Nile Tilapia Oreochromis Niloticus. PLoS ONE.

[28] Tanner H. (1991). Energy Reactions in Atherosclerosis—The Implication of Tricarboxylates in the Pathogenesis of the Disease. Med. Hypotheses.

[29] Aedo J.E., Aravena-Canales D., Valdés J.A., Molina A. (2025). Participation of Membrane-Initiated Cortisol Effects on the Rapid Acclimation of Rainbow Trout (*Oncorhynchus mykiss*) to Increased Salinity. Comp. Biochem. Physiol. A Mol. Integr. Physiol..

[30] Chang R.J.A., Celino-Brady F.T., Seale A.P. (2023). Changes in Cortisol and Corticosteroid Receptors during Dynamic Salinity Challenges in Mozambique Tilapia. Gen. Comp. Endocrinol..

[31] El-Sayed A.F.M. (2006). Current State and Future Potential. Tilapia Culture.

[32] Gorski J., Gannon F. (1976). Current Models of Steroid Hormone Action: A Critique. Annu. Rev. Physiol..

[33] Melamed P., Eliahu N., Ofir M., Levavi-Sivan B., Smal J., Rentier-Delrue F., Yaron Z. (1995). The Effects of Gonadal Development and Sex Steroids on Growth Hormone Secretion in the Male Tilapia Hybrid (*Oreochromis niloticus* × *O. aureus*). Fish Physiol. Biochem..

[34] Yang H., Gao J., Peng X., Han Y. (2024). Application of Synthetic Biology Strategies to Promote Biosynthesis of Fatty Acids and Their Derivatives. Adv. Appl. Microbiol..

[35] Dole V.P., Meinertz H. (1960). Microdetermination of Long-Chain Fatty Acids in Plasma and Tissues. J. Biol. Chem..

[36] Fernandez-Fuente G., Rigby M.J., Puglielli L. (2023). Intracellular Citrate/Acetyl-CoA Flux and Endoplasmic Reticulum Acetylation: Connectivity Is the Answer. Mol. Metab..

[37] Saggerson D. (2008). Malonyl-CoA, a Key Signaling Molecule in Mammalian Cells. Annu. Rev. Nutr..

[38] Park H., Kaushik V.K., Constant S., Prentki M., Przybytkowski E., Ruderman N.B., Saha A.K. (2002). Coordinate Regulation of Malonyl-CoA Decarboxylase, *Sn*-Glycerol-3-Phosphate Acyltransferase, and Acetyl-CoA Carboxylase by AMP-Activated Protein Kinase in Rat Tissues in Response to Exercise. J. Biol. Chem..

[39] Jones P.J.H., Ridgen J.E., Phang P.T., Birmingham C.L. (1992). Influence of Dietary Fat Polyunsaturated to Saturated Ratio on Energy Substrate Utilization in Obesity. Metabolism.

[40] Hers H.G., Hue L. (1983). Gluconeogenesis and Related Aspects of Glycolysis. Annu. Rev. Biochem..

[41] Feng X.-M., Cao L.-J., Adam R.D., Zhang X.-C., Lu S.-Q. (2008). The Catalyzing Role of PPDK in Giardia Lamblia. Biochem. Biophys. Res. Commun..

[42] Chen B., Xiao W., Li D., Zou Z., Zhu J., Yu J., Yang H. (2024). Characterization of Glucose Metabolism in High-Growth Performance Nile Tilapia (*Oreochromis niloticus*). Aquaculture.

[43] Wang Z., Dong C. (2019). Gluconeogenesis in Cancer: Function and Regulation of PEPCK, FBPase, and G6Pase. Trends Cancer.

[44] Castro C., Corraze G., Pérez-Jiménez A., Larroquet L., Cluzeaud M., Panserat S., Oliva-Teles A. (2015). Dietary Carbohydrate and Lipid Source Affect Cholesterol Metabolism of European Sea Bass (*Dicentrarchus labrax*) Juveniles. Br. J. Nutr..

[45] Wade N.M., Skiba-Cassy S., Dias K., Glencross B.D. (2014). Postprandial Molecular Responses in the Liver of the Barramundi, Lates Calcarifer. Fish Physiol. Biochem..

[46] Park J.S., Burckhardt C.J., Lazcano R., Solis L.M., Isogai T., Li L., Chen C.S., Gao B., Minna J.D., Bachoo R. (2020). Mechanical Regulation of Glycolysis via Cytoskeleton Architecture. Nature.

[47] Tanner L.B., Goglia A.G., Wei M.H., Sehgal T., Parsons L.R., Park J.O., White E., Toettcher J.E., Rabinowitz J.D. (2018). Four Key Steps Control Glycolytic Flux in Mammalian Cells. Cell Syst..

